# Non-high-density to high-density lipoprotein cholesterol ratio and its association with infertility in U.S. women: a cross-sectional study

**DOI:** 10.3389/fendo.2024.1451494

**Published:** 2025-01-14

**Authors:** Yajie Zhang, Cong Lu, Lili Li, Hongyu Li

**Affiliations:** Department of Obstetrics and Gynecology, The Third Affiliated Hospital of Zhengzhou University, Zhengzhou, China

**Keywords:** NHHR, NHANES, cross-sectional research, infertility, women

## Abstract

**Objective:**

To investigate the relationship between Non-High-Density Lipoprotein Cholesterol to High-Density Lipoprotein Cholesterol Ratio (NHHR) and infertility in US female adults aged 20 to 45.

**Methods:**

Our research team utilized data from the 2013–2018 National Health and Nutrition Examination Survey (NHANES) to conduct a cross-sectional study. Multivariable logistic regression was conducted to examine the association between NHHR and infertility, with trend tests providing additional insight into this relationship. Further, smoothed curve fitting was applied for a more detailed exploration. To ensure the robustness of our results, we conducted subgroup and sensitivity analyses.

**Results:**

Between 2013 and 2018, our study included 2,947 participants, with 342(11.6%) self-reported infertility. A positive association was found between NHHR and infertility (OR=1.17,95%CI:1.07-1.27). Compared with the first trimester, the third trimester of NHHR was associated with an OR of 1.79(95% CI: 1.31–2.44) in model 3. The results of subgroup analyses revealed that the association between NHHR and infertility was nearly consistent.

**Conclusion:**

NHHR demonstrated a positive correlation with infertility among U.S. female adults. Further investigation is needed to explore their association better and the underlying mechanisms.

## 1 Introduction

Infertility affects thousands of women of reproductive age, with prevalence estimates ranging from 3% to 30% (1, 2). In the United States, the incidence of infertility among women of childbearing age is 15.5%, increasing annually by 0.37% (3). Data from a Chinese multicenter study indicates that approximately 24.58% of women experience infertility (4). Due to its significant impact on human development, the CDC has recommended prioritizing the diagnosis and treatment of infertility (5). Various factors are associated with infertility, including age, obesity, alcohol consumption, smoking, education level, and medical history (6–9). Emerging research indicates that abnormal lipid metabolism may adversely affect female reproductive function (10).

According to the two-cell dual gonadotropin theory (11), altered cholesterol levels can impact the onset and progression of female reproductive diseases by influencing sex hormone activity. Recent multivariate Mendelian randomization research has demonstrated a positive correlation between elevated LDL-C levels and an increased risk of infertility (12). Furthermore, a prospective cohort study indicated that serum lipid concentrations might be associated with reduced fertility and prolonged time to pregnancy in women (13). Another study reported an association between reduced fertility and abnormal lipid levels, including HDL-C, LDL-C, TC, and TG (14). In a nude mouse model of endometriosis, simvastatin, a commonly used lipid-lowering drug, was found to inhibit the proliferation of human endometrial stromal cells and decrease the number and size of endometrial implants, suggesting potential benefits for the treatment of infertility (15).

NHHR, a newly developed lipid profile, serves as an innovative indicator for assessing cardiovascular and cerebrovascular disease risk (16, 17). Recent studies have demonstrated a correlation between NHHR and various conditions such as metabolic syndrome, chronic kidney disease, periodontitis, depression, and suicidal ideation (18–22). However, the relationship between NHHR and female infertility has not been previously investigated. To address this gap, we analyzed data from NHANES 2013-2018 to explore the potential correlation between NHHR and infertility.

## 2 Materials and methods

### 2.1 Participants

For this cross-sectional analysis, data were obtained from the NHANES, which is conducted by the National Center for Health Statistics (NCHS) under the Centers for Disease Control and Prevention (CDC) to assess the nutritional and health status of individuals across the United States. The NHANES protocols received approval from the NCHS Institutional Review Board, ensuring ethical compliance, and informed consent was obtained in writing from all participants. The dataset utilized in this study is publicly accessible on the NHANES website (https://www.cdc.gov/nchs/nhanes/index.html).

**Figure 1:**
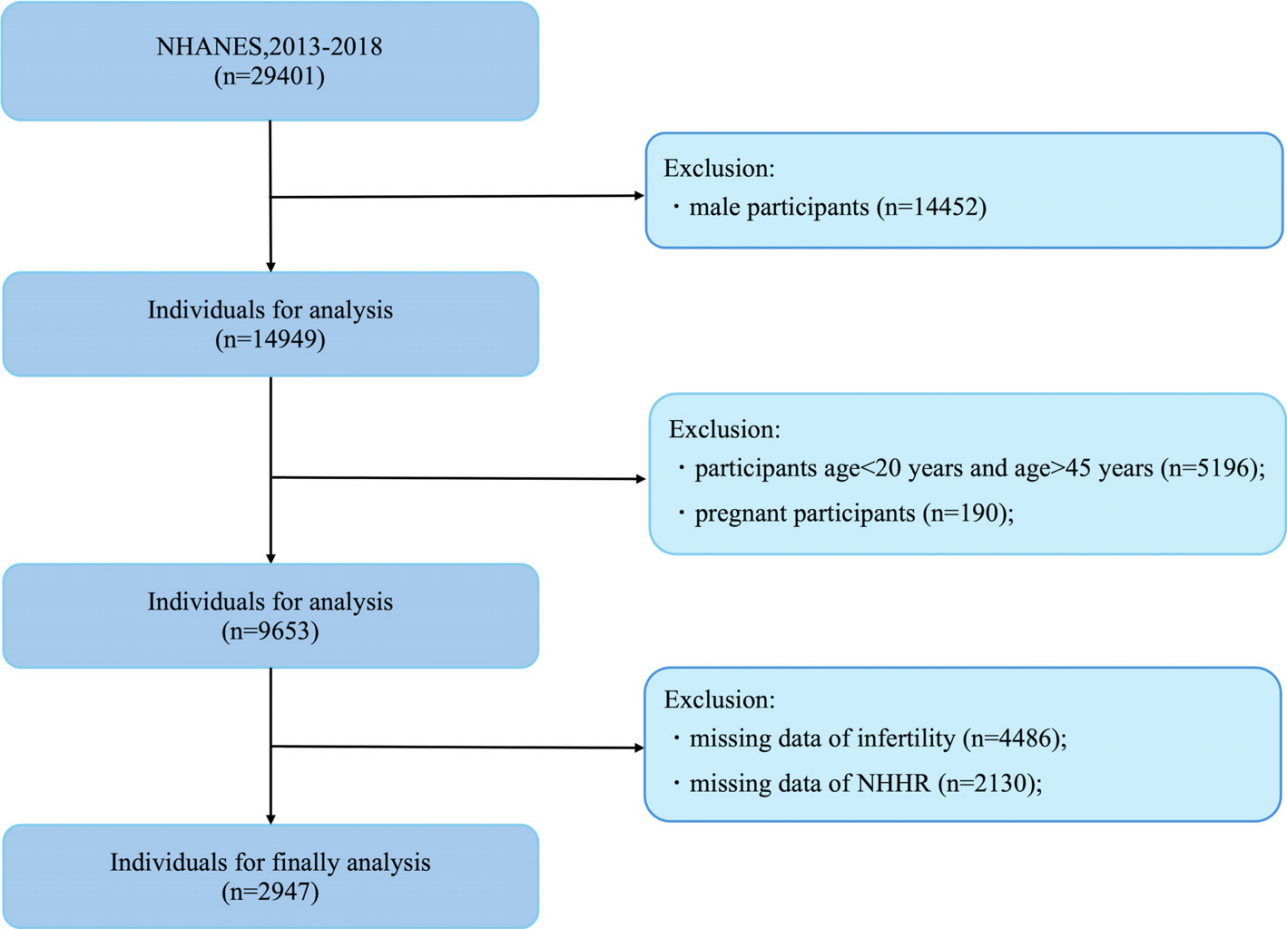
Flowchart of sample selection from the NHANES 2013-2018.

In this study, the cohort was drawn from individuals participating in the NHANES from 2013 to 2018 who had complete records for both the NHHR and Reproductive data. The initial sample consisted of 29,401 subjects. Exclusions were made for male participants(n=14,452), age<20 years and age >45 years participants (n = 5,196), pregnant(n=190), and those lacking data on reproductive (n = 4,486) and NHHR (n=2130), resulting in a final sample of 2,947 participants eligible for analysis (**Figure 1**).

### 2.2 Exposure definitions

NHHR was calculated following the methodologies outlined in previous studies (17). Specifically, NHHR is determined by the ratio of nocturnal heart rate variability to HDL-C, which involves subtracting HDL-C from total cholesterol levels. The concentrations of TC and HDL-C were quantified enzymatically using an automated biochemical analyzer to ensure the accuracy of the measurements.

### 2.3 Outcomes definitions

Infertility is evaluated based on questions from the Reproductive Health Questionnaire (RHQ074): “Have you attempted to become pregnant for at least one year without success?” and “Have you been unable to conceive for at least one year?”

### 2.4 Covariates

In the analysis of NHHR utilization and infertility data, adjustments were made for covariates identified in previous research as influential factors. The demographic characteristics included age and race, with race categorized as Hispanic American, Other Hispanic, Non-Hispanic White, Non-Hispanic Black, and Other. Socioeconomic factors considered were marital status (partnered or single), educational attainment (high school graduate or lower versus college graduate or higher), and the poverty-to-income ratio (PIR). Health-related behaviors were also considered, specifically body mass index (BMI), stratified into <25, 25-30, and ≧30 kg/m², smoking status (determined by an affirmative response to having smoked at least 100 cigarettes in a lifetime on questionnaire SMQ020), and alcohol consumption (identified by consuming at least 12 alcoholic drinks per year on questionnaire ALQ101). Medical conditions such as diabetes and hypertension were adjusted for based on prior diagnosis by a healthcare professional. And covariates related to reproductive health such as age at menarche (determination of age at menarche by responses to the questionnaire RHQ010) and regularity of menstruation (identified by an affirmative response to had regular periods in the past 12 months in RHQ031 questionnaire).

### 2.5 Statistical analysis

Participants were divided into two groups according to their infertility history. Categorical variables were expressed as frequencies (n) and percentages (%), while continuous variables were summarized as means and standard deviations (SD). Differences between the groups were examined using independent samples t-tests for continuous variables and chi-square tests for categorical variables. The study investigated the correlation between NHHR and infertility by stratifying NHHR into three equal tertiles: T1 (0.40-1.79), T2 (1.79-2.70), and T3 (2.70-13.93), with T1 serving as the reference group. We constructed three multivariable logistic regression models to evaluate the specified relationship. Model 1 was unadjusted and served as a baseline. Model 2 incorporated adjustments for age, ethnicity, and educational attainment. Subsequently, Model 3 expanded the covariate set to include the poverty income ratio (PIR), BMI, marital status, alcohol consumption, smoking habits, regularity of menstrual cycles, and the age at menarche. The median values of NHHR for each category were treated as continuous predictors to test for linear trends. The strength of associations was expressed as odds ratios (ORs) with 95% confidence intervals (CIs). Additionally, smoothed curve fitting was employed to explore potential nonlinear relationships between NHHR and infertility. Subgroup analyses were subsequently conducted to assess the consistency of the NHHR-infertility association across different demographic groups.

All analyses were performed with R (version 4.2, http://www.r-project.org) or Empowerstats (version 4.2, www.empowerstats.com, X&Y Solutions Inc., Boston, MA, USA). Statistical significance is defined as two-sided p < 0.05.

## 3 Results

### 3.1 Baseline characteristic

This study included 2,947 participants aged 20 to 45 years, of whom 11.6% (342/2947) were diagnosed with infertility. **Table 1** presents the demographic and clinical characteristics of the cohort. The mean age of infertile women was 35.39 years, compared to 32.76 years for noninfertile women, while the mean BMI was 32.0 kg/m² and 29.42 kg/m², respectively. Infertile women were significantly older and exhibited higher obesity rates compared to their noninfertile counterparts (p<0.001). Furthermore, a higher proportion of infertile women were cohabiting with a partner (72.51% *vs*. 55.70%, p<0.001). Additionally, noninfertile participants demonstrated lower smoking rates (p<0.05) and had reduced TC and NHHR levels (p<0.001).

**Table 1:** Basic characteristics of participants by infertility among U.S. adults.

Characteristics	Total	Infertility		P value
	(N=2947)	No (n=2605)	Yes (n=342)	
Age, years	33.07 ± 7.55	32.76 ± 7.57	35.39 ± 6.94	<0.001
Race, %				0.163
Mexican American	17.54	17.66	16.67	
Other Hispanic	10.28	10.67	7.31	
Non-Hispanic White	33.80	33.21	38.30	
Non-Hispanic Black	21.00	20.88	21.93	
Other race	17.37	17.58	15.79	
Marital status, %				<0.001
Living with partner	57.65	55.70	72.51	
Living alone	42.35	44.30	27.49	
Education level, %				0.825
≤High school	34.75	34.82	134.21	
>High school	65.25	65.18	65.79	
BMI (kg/m^2^)	29.72 ± 8.40	29.42 ± 8.20	32.00 ± 9.25	<0.001
PIR, %				0.397
<1.5	37.26	37.54	35.09	
1.5-3.5	38.14	38.23	37.43	
≥3.5	24.60	24.22	27.49	
Smoke status, %				0.031
Yes	29.22	28.56	34.21	
No	70.78	71.44	65.79	
Alcohol status, %				0.889
Yes	75.16	75.20	74.85	
No	24.84	24.80	25.15	
Regular menstrual cycle, %				0.756
Yes	89.28	89.21	89.77	
No	10.72	10.79	10.23	
Age of menarche, %				0.661
<13	50.93	50.79	52.05	
≥13	49.07	49.21	47.95	
HDL-C (mg/dL)	55.89 ± 15.49	56.22 ± 15.43	53.36 ± 15.67	<0.001
TC (mg/dL)	179.80 ± 34.82	179.21 ± 34.76	184.27 ± 34.97	0.015
NHHR	2.46 ± 1.22	2.42 ± 1.21	2.72 ± 1.29	<0.001

Mean ± SD for continuous variables: the P value was calculated by the linear regression model; (%) for categorical variables: the P value was calculated by the chi-square test.

NHHR, non-High-Density to High-Density Lipoprotein Cholesterol ratio; TC, total cholesterol; PIR, poverty income ratio; BMI, body mass index; OR, odds ratio; CI, confidence interval; Q, quartile; SD, standard deviation.

### 3.2 The relationship between NHHR and infertility

**Figure 2:**
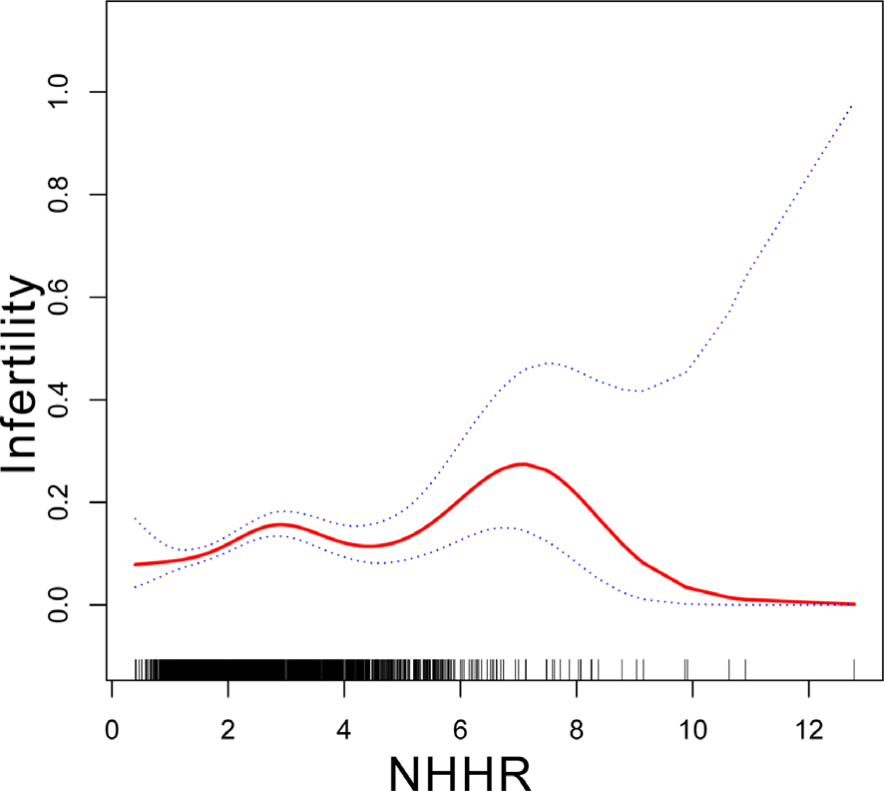
Association between NHHR and infertility. Age, race, education level, PIR, BMI, marital status, alcohol status, smoking status, regular menstrual cycle and age of menarche were adjusted. NHHR, non-High-Density to High-Density Lipoprotein Cholesterol ratio.

**Table 2** displays the association between NHHR and infertility. In the unadjusted model, the OR of NHHR for infertility was 1.19 (95% CI: 1.10–1.29). The findings remained consistent in both the minimally adjusted model (accounting for age, race, and educational level) and the fully adjusted model (considering age, race, education, marital status, PIR, BMI, smoking, alcohol use, regular menstrual cycle, and age of menarche), with ORs of 1.16 (95% CI: 1.07–1.26) and 1.17 (95% CI: 1.07–1.27), respectively. To examine potential nonlinearities, NHHR was converted into a categorical variable by trimester. Following full adjustment, the third trimester of NHHR showed an OR of 1.79 (95% CI: 1.31–2.44) compared to the first trimester (reference). Trend tests indicated statistically significant differences (p for trend <0.001). A smoothed curve fitting was applied to validate the nonlinear relationship further, revealing a positive correlation between NHHR and infertility (**Figure 2**).

**Table 2:** Association between NHHR and infertility.

	Model 1		Model 2		Model 3	
	OR (95%CI)	P value	OR (95%CI)	P value	OR (95%CI)	P value
NHHR	1.19 (1.10,1.29)	P<0.001	1.16 (1.07,1.26)	P<0.01	1.17 (1.07,1.27)	P<0.001
T1	Reference		Reference		Reference	
T2	1.59 (1.18,2.15)	P<0.01	1.56 (1.15,2.11)	P<0.01	1.56 (1.15,2.12)	P<0.01
T3	1.95 (1.46,2.60)	P<0.001	1.80 (1.32,2.43)	P<0.001	1.79 (1.31,2.44)	P<0.001
P for trend		P<0.001		P<0.001		P<0.001

Model 1 adjust for: None.

Model 2 adjusted for age, race, and educational level.

Model 3 adjusted for Age, Race, Educational level, Marital status, PIR, BMI, Smoke status, Alcohol status, Regular menstrual cycle, and Age of menarche.

NHHR, non-High-Density to High-Density Lipoprotein Cholesterol ratio; OR, odds ratio; TC, total cholesterol; PIR, poverty income ratio; BMI, body mass index.

NHHR divided into three equal tertiles: T1 (0.40-1.79), T2 (1.79-2.70), and T3 (2.70-13.93).

### 3.3 Subgroup and interactive analysis of NHHR with infertility

**Figure 3:**
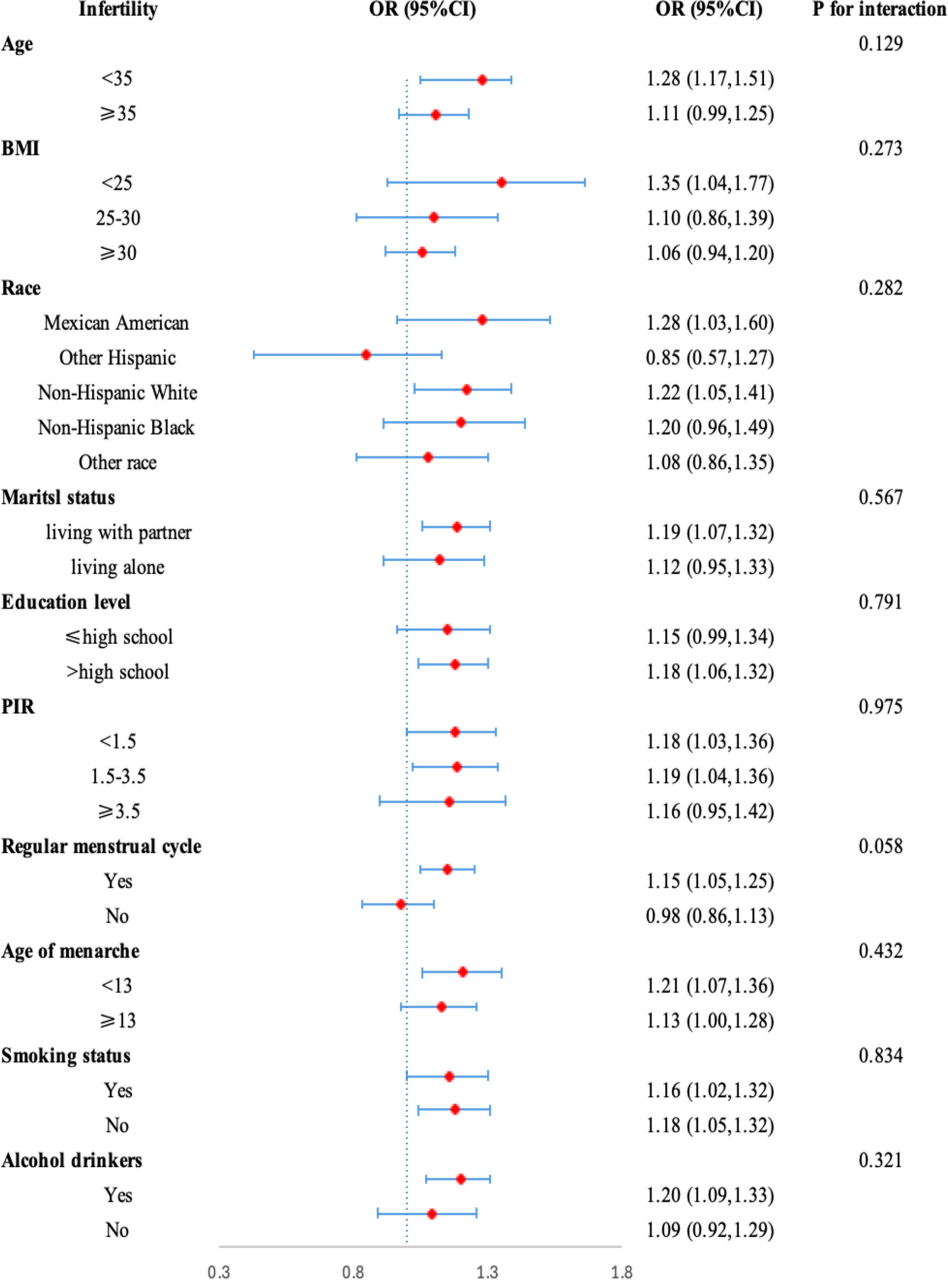
Subgroup analysis of risk factors for the association between NHHR and Infertility. In the subgroup analysis, stratified by age, race, education level, PIR, BMI, marital status, alcohol status, smoking status, regular menstrual cycle, and age of menarche were adjusted, respectively.

We performed the subgroup analysis and interaction tests to ensure the robustness of the relationship between NHHR and infertility among different subgroups. As shown in **Figure 3**, the relationship between NHHR and infertility was nearly consistent among different subgroups of age, BMI, race, marital status, educational level, PIR, smoking status, alcohol status, regular menstrual cycle, and age of menarche. Furthermore, the interaction among different subgroups did not affect the relationship between NHHR and infertility (p for interaction>0.05).

## 4 Discussion

In this study, we stratified participants by age, race, education level, marital status, BMI, PIR, smoking status, alcohol status, regular menstrual cycle, and age of menarche to assess the relationship between NHHR and infertility across varied demographic groups. We found a positive correlation between NHHR and the likelihood of infertility (OR=1.79,95%CI: 1.31-2.44) and this correlation was stable across different subgroups.

NHHR is a novel lipid ratio evaluating atherogenic lipids. Although its role in infertility is unexplored, studies on lipids and female fecundity show mixed results. One trial found that low lipid levels before pregnancy reduced fertility in women with miscarriage history, with higher TG, TC, and LDL-C, and lower HDL-C, increasing infertility risk and prolonging time-to-pregnancy (23). Another trial in PCOS women found increased serum lipids negatively affected reproductive outcomes, with higher LDL-C lowering odds of ovulation, clinical pregnancy, and live births (24). However, Zhu and colleagues identified a significant association between LDL-C and female infertility using both univariate and multivariable two-sample Mendelian randomization (MR) analyses, while HDL-C was found to be irrelevant (12). Similarly, Xu et al. conducted an MR analysis utilizing genetic association data from large GWAS and reported no significant associations between HDL-C, LDL-C, and female infertility (25).

Few studies have explored the underlying mechanisms connecting lipid profiles with infertility. Circulating lipid levels present a double-edged sword: elevated lipid levels can promote oocyte maturation and improve oocyte quality (26). Conversely, long-term exposure to high-fat environments and increased lipid levels in oocytes may induce oocyte toxicity, severely interfering with the meiotic process and ultimately affecting pregnancy outcomes (27). Cholesterol serves as a substrate for ovarian steroidogenesis, playing a crucial role in mammalian hormone levels and the maintenance of pregnancy (28). However, abnormal cholesterol metabolism has been shown to reduce female fertility (29). Arias et al. found that the accumulation of cholesterol and dysfunctional HDL-C in mouse oocytes negatively affects the viability and developmental potential of eggs, leading to reproductive disorders (30). In conclusion, lipids appear to play a significant role in reproductive function. However, the exact mechanisms warrant further investigation.

The principal strength of our research lies in the use of a large, nationally representative sample of adult females in the United States. We conducted comprehensive analyses across various types of variables and incorporated adjustments for covariates to ensure the reliability of our findings. Nonetheless, there are several limitations to consider. First, the cross-sectional study design precludes the establishment of causality between NHHR and infertility, allowing for the possibility of reverse causality due to the bidirectional nature of their relationship. Second, lipid profiles were assessed only once during the study, which may be subject to variations caused by acute stress and random factors. Third, although adjustments were made for numerous confounders, the influence of unmeasured or unknown confounders on the study results cannot be entirely excluded. Ultimately, the ascertainment of infertility within this investigation was predicated on participant-reported data, which is inherently contingent upon the accuracy of individual recall and self-assessment. This approach could potentially engender discrepancies in the documentation of infertility diagnosis and its chronicity, thereby posing a risk of introducing bias into the study outcomes.

## 5 Conclusion

In conclusion, our results indicate a positive association between NHHR and infertility in US female adults. Future research should include prospective and randomized controlled studies to validate these findings. Additionally, further exploration of the pathophysiological mechanisms underlying these associations is essential.

## Data Availability

The datasets presented in this study can be found in online repositories. The names of the repository/repositories and accession number(s) can be found in the article/supplementary material.
